# Adolescent idiopathic scoliosis: Retrospective analysis of 235 surgically treated cases

**DOI:** 10.4103/0019-5413.58604

**Published:** 2010

**Authors:** Ranjith Unnikrishnan, J Renjitkumar, Venugopal K Menon

**Affiliations:** Department of Orthopedics, Amrita Institute of Medical Sciences, Kochi, Kerala, India

**Keywords:** Adolescent idiopathic scoliosis, spinal deformity, surgery

## Abstract

**Background:**

The surgical treatment of adolescent idiopathic scoliosis (AIS) has taken great strides in the last two decades. There have been no long term reported studies on AIS from India with documented long term followup. In this study we review a single surgical team's series of 235 surgically treated cases of AIS with a follow-up from two to six years.

**Materials and Methods:**

Pre operative charts, radiographs and MRI scans for 235 patients were collected for this study. The patients were grouped into three groups where anterior correction and fusion (n=47), posterior correction and fusion (n=123) and combined anterior release and posterior instrumentation (n=65) was performed. Each group was divided into two subgroups based on the surgical approach and instrumentation strategy (all screw construct or hybrid construct) used. Patients were followed up for coronal and saggital plane corrections, apical vertebral translation (AVT), trunk balance and back pain. The percentage of correction was calculated in each group as well as sub groups.

**Results:**

The incidence of MRI detected intraspinal anomaly in this series is 5.9% with 3.4% of them requiring neurosurgical procedure along with scoliosis correction. Average coronal major curve correction was 66% in the all screw group and 58.5% in the hybrid group. The coronal plane correction was better when the all screw constructs were employed. Also, the AVT and trunk balance was better with the all screw constructs. The anterior corrections resulted in better correction of the AVT and trunk balance as compared to the posterior correction. There were eight (3.4%) complications in this series. The coronal and saggital plane correction paralleled the published international standards.

**Conclusion:**

The coronal plane correction was better when all screw constructs were employed. Use of all pedicle screw systems obviated the need for costoplasty in most cases. The increased incidence of intraspinal anomaly may warrant a routine pre operative MR imaging of all adolescent scoliosis needing surgical treatment.

## INTRODUCTION

Idiopathic scoliosis is a diagnosis of exclusion made after a patient has been evaluated for underlying congenital, syndromic, or neurologic causes. Approximately 80% of curves are ultimately diagnosed as idiopathic.^1^ The prevalence of adolescent idiopathic scoliosis (AIS) (age at diagnosis above 10 years) is estimated at approximately two to three per cent of school going children, however, the prevalence decreases with increasing curve severity.^2^ There is an overwhelming female preponderance for curves of larger magnitudes with a ratio of 9:1 in curves larger than 40°.^2^–^4^ Treatment of AIS may be observational, bracing, or surgical. Observation may be indicated in curves less than 25° in skeletally immature patients and for curves up to 45° to 50° in skeletally mature patients. Bracing is considered in skeletally immature patients with curves between 25° and 40°. Surgical treatment is considered in patients with progressive curves greater than 40° that fail or cannot tolerate bracing and those with curves greater than 45° at skeletal maturity.^3^ The treatment of scoliosis in our series was paralleling the developments in surgical techniques and the availability of implant systems locally along with mandatory preoperative imaging study of the patients. In this study, we review a series of 235 surgically treated cases with two to six years follow-up.

## MATERIALS AND METHODS

Out of a total 495 spinal deformity procedures documented in the hospital register, performed between 2000 and 2007, complete documentation and follow-up results were available for 302. Cases of congenital deformities (n=37), neurofibromatosis (n=12), juvenile scoliosis (n=16) and neuromuscular scoliosis (n=2), were excluded from the present study group. The data for 235 adolescent idiopathic scoliosis patients were collated and retrospectively analyzed. The demographic (age, sex, age at menarche, family history, urban/rural house hold) and anthropometric data (standing, sitting height, arm span) was obtained. Back pain was assessed with the visual analog scale (VAS) score in all patients pre operatively and at six months post-op. All patients underwent whole spine standing anteroposterior (AP) radiographs, lateral radiograph, supine bending films to assess the curve flexibility. The pre and postoperative images were analyzed for the following parameters; Lower end vertebra (LEV), Upper end vertebra (UEV), Lower instrumented vertebra (LIV), Upper instrumented vertebra (UIV), distal stable vertebra, Cobb's angle (UEV to LEV), instrumented levels, coronal C7-Central Sacral Vertical Line (CSVL), apical vertebral translation (AVT), trunk shift, T5-T12 saggital, T12-S1 saggital, proximal junctional kyphosis, distal junctional kyphosis and Risser index. The measurements were done on PACS software, Amrita Med Vision (Amrita Technologies).

**Figure 1:**
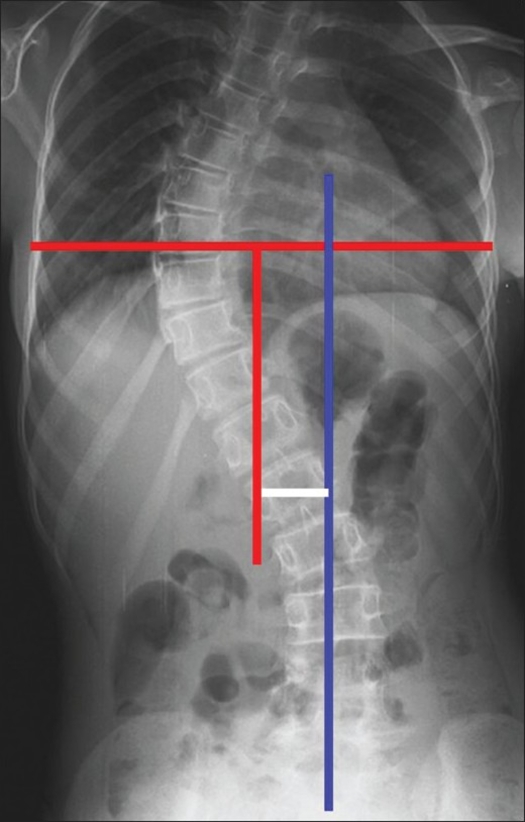
Measurement of trunk balance: X-ray of dorsal, lumbar spine including lumbosacral junction (anteroposterior view) showing a central sacral line (blue), vertical line (red) bisecting a line drawn from the peripheral edges of the ribs of the apical vertebra. The horizontal distance between the vertical lines (white) quantify the trunk imbalance

Iliac crest ossification (Risser Index) has been routinely documented in all our cases. The thoracic apical vertebral translation was measured as the horizontal distance measured from the C7 plumbline to the midpoint of the apical vertebra/disc space; the distance for the lumbar AVT is measured from the central sacral line. To measure trunk balance, two vertical lines are drawn on a standing AP radiograph. The first line is the central sacral line and the second vertical line bisects a line drawn from the peripheral edges of the ribs of the apical vertebra. The horizontal distance between the vertical lines quantify the trunk imbalance^6^ [Figure 1]. This was repeated at three months, six months and then yearly. All the patients underwent pulmonary function test pre operatively and at three, six and 12 months postoperatively. The whole spine was screened by MRI in all patients as a routine pre operative imaging study. Indications for surgery was curve over 40° in children with significant growth remaining (Risser index, 0-2 and pre menarcheal) and curves over 50° in the skeletally mature child (especially in the lumbar spine) and clinically significant coronal plane decompensation of the spine.^3^,^4^ No standardized outcome instrument was used till 2004 (n=110); from 2005 (n=135) onwards the SRS 24^5^ was administered pre operatively and at every follow up visit. The classification system employed was as described by King and Moe^6^ (n=110)till 2004. From 2005 onwards the scheme included the Lenke classification^7^ (n=135)as well as the Kings types.

The instrumentation strategy employed was based on the strategic vertebrae as described by Cotrel and Dubousset^8^ when posterior spinal fusion was elected. All the lumbar (Lenke Type V) and a few thoracolumbar curves were treated by anterior correction and fusion. For thoracolumbar curves this decision was based on fusion levels limited to less than seven vertebrae and at least 50% correction on side bending radiographs. Thus, anterior correction and fusion was employed in 47 patients (group A). In 12 patients of group A (group A.1), the Hall protocol of limited instrumentation with overcorrection^9^,^10^ was employed and in the remaining 35 (group A.2) the UEV and LEV were chosen as end points of fusion. Posterior correction and fusion was performed in 123 patients (group B). The group B patients were further subdivided into group B.1 (n=42), where hybrid instrumentation constructs was employed and group B.2 (n=81) where all pedicle screw instrumentation was employed. The decision to use hybrid constructs and pedicle screw systems were based upon the availability of them locally. Combined anterior release and posterior instrumentation was performed in 65 patients (group C) which was also further subdivided into C.1 (hybrid constructs, n=28) and C.2 (all screw constructs, n=37). The indications for an anterior release were

1. Very stiff curves(> 75°) correcting to less than 30°.
2. Premenarchal patient, age <11 years.
3. Risser grade 0-1.

**Figure 2:**
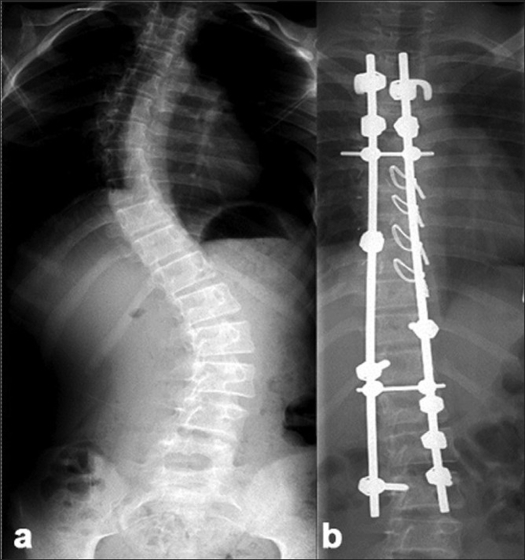
(a) Preoperative X-ray of dorsolumbar and sacral spine (anteroposterior view) of a 12 year old female with Lenke Type1C curve. (b) Post-operative radiograph depicting hybrid constructs with screws at the bottom and hook-claws at the top and sublaminar wires at the concave apex, resulting in a good correction

**Figure 3:**
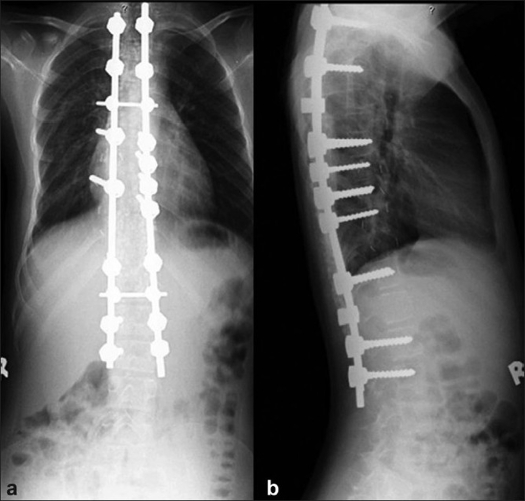
Post-operative anteroposterior (a) and lateral (b) radiographs of dorsal and lumbar spine depicting the deformity correction achieved by all screw construct

**Figure 4:**
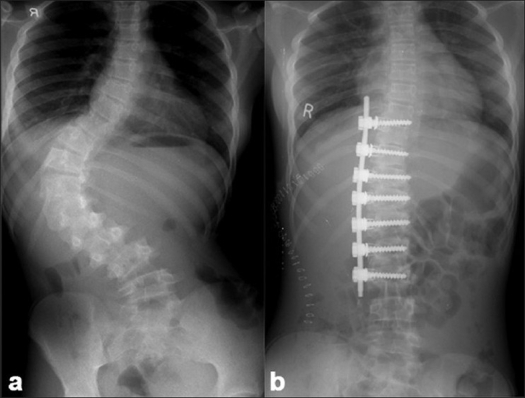
(a) Pre-operative X-ray of dorsal lumbar and sacral spine (anteroposterior view) of a 14 yr old female with Lenke Type 5C right sided thoracolumbar curve. Post-operative radiograph depicting good correction achieved

The instrumentation systems employed matched the evolution of the various systems and their availability in the country. Hybrid constructs (Hook, screw, sublaminar wires) were employed in 70 patients [Figure 2] and 118 patients received all pedicle screw instrumented corrections [Figure 3]. As the expertise in accurately inserting pedicle screws into the deformed thoracic pedicle improved, more cases were corrected employing the “all screw” constructs. The 47 anterior corrections utilized the screw and staple system with single rigid rod constructs [Figure 4]. The correction maneuvers employed were transverse translation with rod rotation in the earlier hybrid systems which were later on adapted to a combination of transverse translation, rod rotation and direct vertebral rotation in the “all pedicle screws” constructs. Convex side compression is additionally performed in anterior corrections and posterior corrections of lumbar curves. Segmental derotation was performed for transverse plane correction in all cases where an appropriate implant construct allowed it. Thoracoplasty was performed electively in 198 patients (since we regularly use rib grafts for fusion and rarely was iliac crest graft harvested) and later on it was performed only if deemed necessary (3 out of the 37 cases), after corrective maneuvers; this change in strategy was more so after the introduction of all pedicle screw systems.

**Figure 5:**
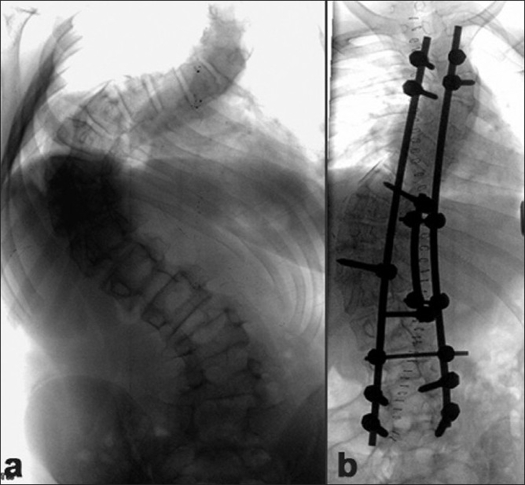
(a) Pre-operative X-ray of dorsal, lumbar spine including lumbosacral junction (anteroposterior view) of a 15 year old female with rigid Lenke Type 4C curve having a major Cobb's angle is 104° (b) post-operative radiograph showing correction achieved with use of a three rod technique. Postoperative major Cobb's is 48°

Three patients with very large and stiff curves (over 100°) were treated with a three-rod technique [Figure 5] in addition to an anterior release. Stagnaras' wake-up test was utilized as the sole modality in 70 patients and subsequent patients (n=165) had a combination of wake up test and intra operative somatosensory evoked potentials (SSEP) monitoring, when the facility became available to us. A total of 201 patients had autograft sourced from the ribs resected at thoracoplasty mixed with synthetic ceramic bone substitute in the ratio 1:1. In 34 patients autograft was harvested exclusively from the spinous processes, and laminae of the segments to be fused. Epidural analgesia was routinely used for post-op analgesia.^11^ Our postoperative strategy has been to ambulate the patient with a light weight TLSO brace when hook/ wire devices (n=70) are used and without brace when all screw constructs (n=165) are used. Ambulation starts on the third postoperative (PO) day and the patient is discharged between days five and 14. The percentage of correction was calculated in each group as well as sub groups. Comparative analysis was not performed in between groups because of the non uniformity of the curve type, severity and flexibility in each group and sub group, as well as the non randomization of the instrumentation and correction strategies employed. Results from this series with respect to pulmonary function test and rib generation have been published.^12^,^13^

The latter part of this series includes a comparison of anterior versus posterior approach in Lenke Type 5 C (Thoracolumbar/Lumbar) curves. The results of this subgroup are being followed up as part of another ongoing study with respect to curve correction, fusion levels, complications, trunk balance, apical vertebral translation (AVT) and outcome measures.

## RESULTS

The age of patients varied from 11 to 19 years (Mean: 13.2yrs). There were 42 male and 193 female patients. The 42 male patients were of normal body habitus. Out of the 193 female patients,110 were tall for their age and thin as determined from the growth charts^14^ and 83 were of normal body habitus. The incidence of intraspinal anomalies like syrinx (n=12), Chiari malformation (n=8) in this series based on the MRI screening is 5.9% (total of 14 patients). Of these 14 patients, eight (3.4%) underwent neurosurgical procedure prior to the scoliosis correction. This incidence of intraspinal anomaly warranting a surgical procedure (3.4%) is higher than earlier reports.^6^ There were a total of eight complications (3.4 %) including one death (following a vascular injury after anterior surgery) and three cases of neurologic deficits, two of which were detected during the wake up test and had the implants removed and in-situ fusion performed intra-operatively. Both these patients recovered by sixth postoperative month and the third patient had a partial recovery (normal muscle power but residual spasticity in one lower limb); yet they were happy with the outcomes based on the SRS 24. There were three cases of infection of which, one patient settled with multiple debridements, two patients had their implants removed after fusion. There is one case of pseudoarthrosis (large curve from group C.1) who is awaiting a revision surgery. The other factors which resulted in poor patient satisfaction were postoperative shoulder imbalance (three cases). There were no re operations for these 11 patients yet at the time of this writing. Analysis of the different sub groups was done to assess the coronal plane and sagittal plane balance.

The VAS score for pain was average of 3.5 in 15.3% (n=36) pre operatively. The immediate postoperative pain was not assessed as the post operative discomfort might skew the results. The VAS assessment of pain at six months postoperative was average of 0.08 with one patient reporting no change of back pain after surgery. The major curve correction was assessed by the pre op and postoperative Cobb measurements in single major curves. In double major and triple major curves the Cobb's angle of curves were averaged and arithmetic mean used for comparison [Table 1].

**Table 1:** Mean coronal plane data

	Cobb's (Deg) preoperative mean (range)	Cobb's (Deg) post operative mean (range)	% correction	Final Cobb's (Deg)	Loss in deg
A.1	42 (38-56)	10 (8-14)	76	13.8	3.8
A.2	52 (40-62)	8 (6-12)	84	10.3	2.3
B.1	46 (38-64)	12 (8-20)	58	13.8	1.8
B.2	48 (40-89)	14 (11-24)	70	15	1.0
C.1	57 (48-64)	23 (18-26)	59	24.2	1.2
C.2	54 (46-74)	20 (12-28)	62	20.8	0.8

The average coronal Cobb's angle correction was 76% in the A1 and 84% in the anterior group. This sub group, especially the A.1 showed the largest loss of correction (3.8°) in comparison to the other groups. The coronal Cobb's angle correction in group B and group C showed similar trend with all posterior screw constructs showing the highest (70%) correction. The saggital Cobb's angle correction in the lumbar and thoracolumbar sub groups (group A.1 and A.2) showed a similar trend of increasing lordosis, with the A.2 group patients showing an average increase of 3° more than the Hall sub group A-1. The saggital Cobb's angle correction in the all posterior(B) and combined anterior and posterior group (group C) showed an improvement in kyphosis, with the hybrid constructs producing a 3° more increase in correction as compared to the all pedicle screw constructs. There were three cases of proximal junctional kyphosis (PJK) in the A.1 (Hall) sub group, defined as more than 10° of saggital angulation between the proximal instrumented vertebra and two levels proximal to it. No other patient showed a significant PJK. The distal junctional kyphosis measured from the LIV and two levels caudal to it, of more than 4° were present in total of eight patients [Table 2].

**Table 2:** Saggital plane data

	Preoperative deg mean (range)	Postoperative deg mean (range)	PJK (No. of patients)	DJK (No. of patients)
A.1 (T12-S1)	−47 (−20 to −56)	−50 (−42 to −60)	3	1
A.2 (T12−S1)	−50 (−18 to −61)	−56 (−46 to −62)	0	0
B.1 (T5-T12)	22 (12 to 33)	36 (30 to 42)	0	2
B.2 (T5-T12)	20 (10 to 38)	33 (28 to 36)	0	2
C.1 (T5-T12)	20 (8 to 36)	35 (30 to 42)	0	1
C.2 (T5-T12)	24 (12 to 30)	33 (28 to 40)	0	2

PJK: proximal junction kyphosis, DJK: distal junction kyphosis

The balance parameters were computed as tabulated [Table 3]. In double major and triple major curves the AVT was averaged for each patient and the arithmetic mean calculated as the AVT for that particular patient and the same was repeated at the follow up visits. Truncal imbalance was higher in the Group A patients with AVT of 46 and 48 mm pre operatively and this group also showed the largest improvement in the AVT post operatively. This group of patients also had the highest improvement of C7 plumb line. Group B and C patients had moderate improvement of the truncal imbalance, and did not show a great change in between sub groups [Table 3].

**Table 3:** Balance parameters

	AVT preop mm mean	AVT postop mm	Translatn (mm)	C7 plumb preop (mm)	C7 plumb postop (mm)	Change (mm)	Trunk shift (mm) preop	Trunk shift (mm) postop	Change mm
A.1 (n = 12)	46	12	34	30	17	13	40	10	30
A.2 (n = 35)	48	8	40	32	8	24	42	12	30
B.1 (n = 42)	24	14	10	25	20	5	28	20	8
B.2 (n = 81)	22	10	12	29	22	7	26	16	10
C.1 (n = 28)	33	22	11	29	24	5	24	19	5
C.2 (n = 37)	36	24	12	26	22	4	22	10	12

AVT = Apical vertebral translation

The coronal plane correction was better when the all screw constructs were employed. Also the AVT and Trunk balance was better with the all screw constructs. The anterior corrections resulted in better correction of the AVT and trunk balance as compared to the posterior correction [Table 3]. Sub group analysis or statistical test of significance was not applied to any of the parameters, as the groups were not matched for curve type, severity and instrumentations employed. Statistical inference from this diverse unmatched cohort may not be relevant, and that was not the intention of this study. Functional outcomes were not assessed as the SRS 24 scores were not available for the entire study group as it was instituted late in to the study with minimum follow-up of two years and maximum follow-up of nine years (Mean 3.8 years).

## DISCUSSION

The value of Iliac apophysis (Risser index) as an indicator of growth and maturation has been debated.^15^–^18^ We have used it as one of the factors that help us make surgical decisions. Children who are Risser 0 (especially when premenarchal and less than 11 years) were considered for additional anterior fusions to prevent crankshaft phenomenon; (some of these concepts have slowly changed with our own evolution through the three-dimensional correction systems and all screw instrumentation.) Several authors have suggested that the triradiate cartilage of the acetabulum, the wrist and hand ossification centers and the vertebral apophysis itself be used as growth markers. But we have not changed from the use of Risser grades. Additional growth and maturity parameters like Tanner's tables are not applicable in our very culturally sensitive environment.

The presence of pain is another intriguing finding in scoliosis. Approximately 30% patients are documented to have pain.^19^ The pattern is very nonspecific and uncharacteristic of major spine pathology. In our series, 36 (15.3%) patients reported pain which greatly improved post operatively in all but one patient.

The parameters studied showed results paralleling the published international standards. Average coronal major curve correction was 66% in the all screw group and 58.5% in the hybrid group.^20^–^25^ Anterior and posterior instrumentation had differential effects on the saggital plane in patients with adolescent idiopathic scoliosis. However, the overall magnitude of the differences was small. Properly performed, both approaches can result in acceptable saggital profiles.^26^ This was evident from the relatively similar amount of correction among the different instrumentation systems used in this series. The detailed analysis between curve types was not undertaken due to reasons cited earlier. Currently, anterior or posterior instrumentation can be used in the treatment of selected idiopathic curves. Proponents of anterior surgery cite the ability to save distal fusion levels, achieve greater correction, and avoid spinal extensor muscle disruption. Advocates of posterior fusion note its proven reliability, avoidance of chest cage disruption, and ability to better control shoulder imbalance.^27^ Thoracic pedicle screws also resulted in superior rotation and thoracic torsion correction and affords potentially less perioperative, particularly pulmonary, morbidity.^28^ This enhanced correction enabled by the thoracic screws probably explains the lesser need of costoplasty in our “all screw” cases and hence lower morbidity and early recovery from surgery. The statistical claim for this can only be substantiated with a long term follow up with a larger series of patients.

The change in the AVT and trunk balance was better with the all screw constructs. This probably reflects the better transverse plane correction facilitated by the fixation of all three spinal columns by the pedicle screws. In group A.1 where Hall's principle was employed for short anterior correction, the follow up radiographs revealed significant proximal junction kyphosis^29^,^30^ in three patients. This, along with the adding on of vertebrae to the curve in our series, led us to revert back to the “End to End” fusion in anterior instrumented corrections.

The complications in this series are well with in the published guidelines, with an exceptionally low incidence of pseudoarthrosis. The reason for this is believed to be the meticulous preparation of the graft bed and the facet fusion employed in every posterior correction. The rate of re-operation is low as compared to the published reports.^31^ However, this may not be the true incidence as three patients with prominent posterior implants are not willing to be re operated as they report that the implants are not affecting them and they are quite happy with the result.

There are many limitations of this study. This is a retrospective review. An outcomes instrument was not used consistently to allow comparisons at pre- or postoperative intervals in the former half of cases. Subgroup analysis was not anticipated during the enrollment period of the study. The sub groups could not be age matched or grouped as per the curve type classification and severity owing to the retrospective nature as well as the instrumentation strategy could not be tested among similar curve types. The averaging of major and minor curve parameters may have induced errors in forming any definite conclusions regarding the instrumentation systems involved, which is not the intention of this analysis.

## CONCLUSION

This study represents a longitudinal study of a pathology treated and followed-up by one team of surgeons; the use of all pedicle screw systems has enabled better correction and less use of costoplaty in posterior fusions. Anterior surgery is reserved for thoracolumbar and lumbar curves with excellent restoration of spinal balance. Further follow-up of this cohort is in process with curve classification based analyses, which may provide more clear recommendations with respect to our patient population and implant systems available.
